# Barberry's (*Berberis integerrima*) ingredients suppress T-cell response and shift immune responses toward Th2: an *in vitro* study

**DOI:** 10.4155/fso.15.49

**Published:** 2015-11-01

**Authors:** Shirin Fateh, Shaghayegh Pishkhan Dibazar, Saeed Daneshmandi

**Affiliations:** 1 Department of Immunology, Faculty of Medical Sciences, Tarbiat Modares University, Tehran, Iran

**Keywords:** barberry extract, *Berberis integerrima*, immune response, lymphocyte, MTT, splenocytes

## Abstract

**Aim:**

Food and medicinal applications of barberry date back to 2500 years ago. This study investigates *Berberis integerrima* impact on lymphocytic immune responses.

**Materials & methods:**

Balb/c splenocytes were treated by 0.001–1000 μg/ml of *B. integerrima*aqueous and alcoholic extracts in presence of phytohemagglutinin and lipopolysaccharide mitogens. Cell proliferation was assayed and cytokines were measured using ELISA.

**Results:**

Both extracts suppressed proliferation of phytohemagglutinin stimulated splenocytes (as T cells), while alcoholic extract induced expansion of lipopolysaccharide activated cells (as B lymphocytes) and unstimulated cells (p < 0.05). Both barberry extracts suppressed IFN-γ production (p < 0.05) and enhanced IL-4, IL-10 and TGF-β release from splenocytes (p < 0.05).

**Conclusion:**

Both extracts could suppress T-cell and enhance B-cell proliferation and shift immune responses toward Th2.

## Abstract (lay abstract)

Food and medicinal applications of barberry date back to 2500 years. This study investigates *Berberis integerrima's* impact on lymphocytic immune responses. Balb/c splenocytes were treated by water and alcoholic extract of barberry which have changed cytokine release from cells. Both extracts could suppress T-cell expansion and enhance B-cell proliferation and also shift immune responses toward Th2. Therefore, *B. integerrima* contents can promote humoral immunity and would have beneficial applications in disease and conditions that humoral immunity needs to get stronger.

**Figure 1.**
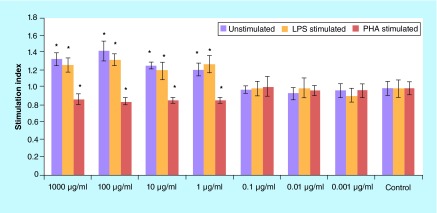
**The values (mean ± standard deviation) of proliferation assay of lypopolysaccharide/phytohemagglutinin/un-stimulated spelnocytes treated with various concentrations of *Berberis integerrima* alcoholic extract.** 1–1000 μg/ml of extracts reduced PHA stimulated splenocytes (as T cells) proliferation and enhanced proliferation of LPS stimulated (as B cells) or unstimulated splenocytes. Significant differences are designated as *p < 0.05. LPS: Lypopolysaccharide; PHA: Phytohemagglutinin.

**Figure 2.**
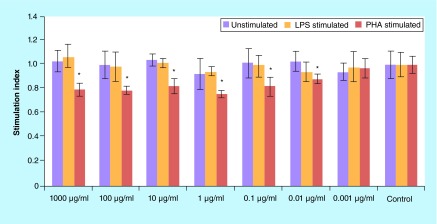
**The values (mean ± standard deviation) of proliferation assay of lypopolysaccharide/phytohemagglutinin/un-stimulated spelnocytes treated with various concentrations of *Berberis integerrima* aqueous extract.** 0.01–1000 μg/ml of extracts reduced PHA stimulated splenocytes (as T cells) proliferation. Significant differences are designated as *p < 0.05. LPS: Lypopolysaccharide; PHA: Phytohemagglutinin.

**Figure 3.**
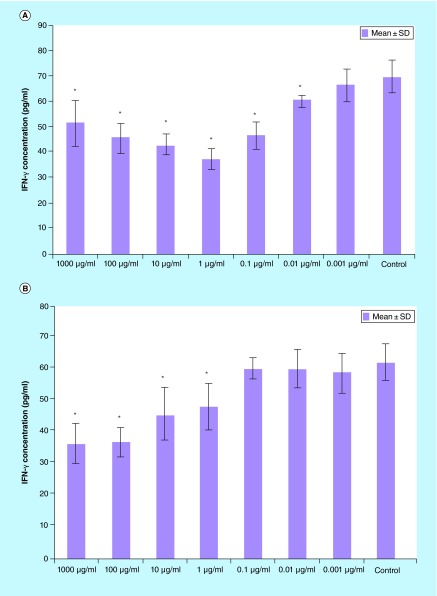
**IFN-γ production in splenocytes treated with various concentrations of *Berberis integerrima* (A) alcoholic and (B) aqueous extracts.** Significant differences were designated as *p < 0.05. SD: Standard deviation.

**Figure 4.**
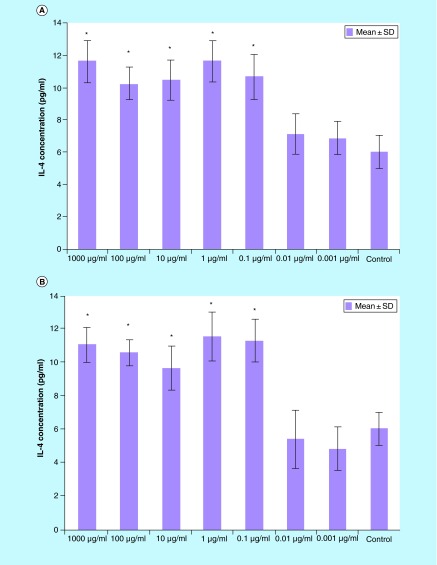
**IL-4 production splenocytes treated with various concentrations of *Berberis integerrima* (A) alcoholic and (B) aqueous extracts.** Significant differences were designated as *p < 0.05. SD: Standard deviation.

**Figure 5.**
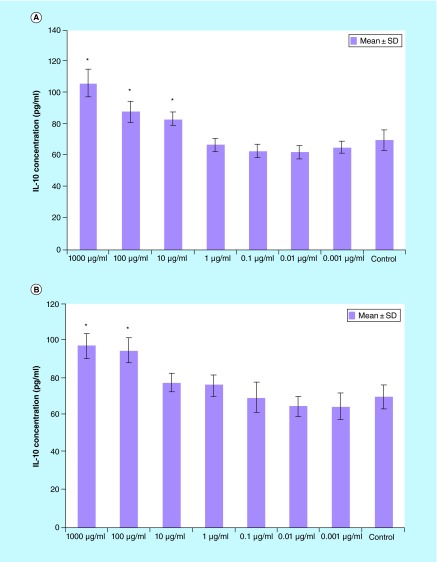
**IL-10 production splenocytes treated with various concentrations of *Berberis integerrima* (A) alcoholic and (B) aqueous extracts.** Significant differences were designated as *p < 0.05. SD: Standard deviation.

**Figure 6.**
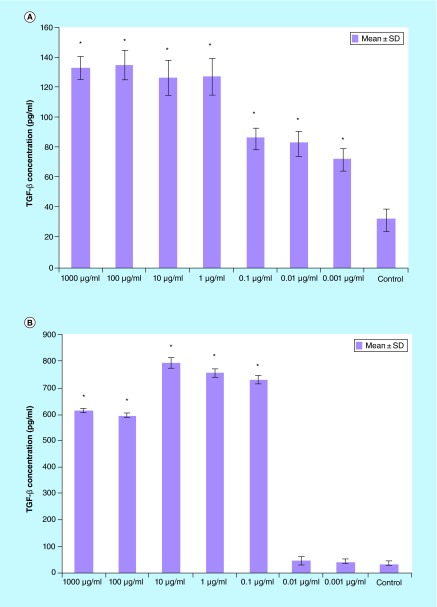
**TGF-β production of splenocytes treated with various concentrations of *Berberis integerrima* (A) alcoholic and (B) aqueous extracts.** Significant differences were designated as *p < 0.05. SD: Standard deviation.

## Background

The roots and barks of Berberis species *(Berberidaceae)* are worldwide used in traditional medicine for the treatment of various ailments [1]. Medicinal use of barberry dates back to more than 2500 years. Herbal active components have different mechanisms of action; finding these components and their mechanisms in the immune system is noticeably significant [2]. Some of herbal drugs and mediators functions are characterized by their influence on lymphocytes. Barberry is an evergreen shrub that belongs to Berberis species (Berberidaceae family) [3,4]. It has yellow-to-brown bark, red-colored fruits, and thick and woody roots covered with a brittle bark [5,6]. The shrub is native to central and southern Europe, northwest Africa and western Asia; it is cultivated for its fruits in many countries. They are edible but very sour, and rich in vitamin C and widely used in cooking [7]. Barberry has been used in Indian folk medicine to treat diarrhea, reduce fever, improve appetite and relieve upset stomach. Today, it is used extensively for medicinal purposes in Iran, including biliary disorders (such as gallbladder disease) and heartburn [8]. Some barberry components which are liable for this plant's effects are alkaloids, such as berberine, oxyacanthine, berbamine and palmatine [9]. Various beneficial effects of Berberine have been studied on cell cycle arrest, apoptosis induction and anti-inflammation. Berberine exhibits antiangiogenesis, anti-invasion and antimetastasis effects on some cancer cell lines [10]. Immunomodulatory function of berberine in enhancing progression of diabetes type one in mice has been shown. Also berberine reduces Th17 and Th1 cell differentiation and cytokine production [11]. Although biological and therapeutic effects of barberry have been reported, its effect on the immune system is not properly investigated. These studies in traditional and current medicine suggest that barberry has diverse effects on the immune system. In this study effects of aqueous and alcoholic barberry extracts were investigated on Balb/c splenocytes, as T and B cells are the major effector cells in cellular and humoral immune responses. Splenocytes from Balb/c were separated into mitogen stimulated (PHA on T cells and lipopolysaccharide [LPS] on B cells) and none stimulated groups. Also the effect of barberry extract on IFN-γ, IL-4, IL-10 and TGF-β secretion was assessed by using BALB/c spleen lymphocytes.

## Materials & methods

### Animals

The study was performed on 6–8-week-old female inbred Balb/c mice, which were purchased from the Pasteur Institute of Iran. Animals were housed in the animal house of Tarbiat Modares University under a 12/12-h light/dark cycle at 24 ± 2°C and had free access to standard mouse chow and sterilized water. All animal experiments were carried out in accordance with Tarbiat Modares University Ethical Committee Acts.

### Plant material

Fruits of *B. integerrima* were collected from plants cultivated in Medicinal Plants Research center, 25 km north of Tehran, Iran, and confirmed by the Agricultural Research Center, Tehran, Iran.

### Preparation of extracts

*B. integerrima* dried fruit was powdered by a mechanical grinder. For alcoholic extract the powder (25 g) was soaked in 100 ml of 96% ethanol, and the solution was mixed on a rotary for 48 h. The mixture was subsequently filtered with Whatman No. 1 filter paper and was lyophilized at -20°C.

For aqueous extract the powder (25 g) was macerated in 100 ml distilled water and the solution was mixed on a rotary for 48 h. The mixture was subsequently filtered with Whatman No. 1 filter paper and was lyophilized at -20°C. Stock of 100 mg/ml was prepared for both extracts, styled using 0.22-μm filters and used in the assays.

### Preparation & treatment of splenocytes

Mice were sacrificed with mild diethyl ether anesthesia. On a clean dissection board under sterile condition the spleen was rapidly excised and was placed into the cell strainer. Tissue was subsequently homogenized in a glass homogenizer. To discard the cell strainer, 10 ml cold Roswell Park Memorial Institute medium (RPMI) 1640 (Sigma Chemical Co, Perth WA) was added to tissue. To obtain a homogeneous cell suspension, homogenized spleen tissues passed through a sterile fine steel mesh. To lyses erythrocytes, 0.75% NH4Cl in Tris buffer (0.02%, pH = 7.2) was added to homogenized tissue and it was centrifuged (360 × g at 4°C for 10 min); the cell pellet was washed three times with phosphate-buffered saline (PBS) and resuspended in RPMI 1640 complete medium supplemented with 11 mM sodium bicarbonate, 2 mM l-glutamine, 100 U/ml penicillin, 100 μg/ml streptomycin and 10% fetal bovine serum. Cell count and viability check was performed using trypan blue and a hemacytometer. Total cell viability was more than 95%. Splenocytes (5 × 10^6^ cells/ml) were cultured into each well of a 96-well flat-bottom microtiter plate (Nunc) in complete medium and PHA (final concentration of 5 μg/ml), LPS (final concentration of 10 μg/ml) or PBS was added to the wells. Barberry ingredients (final concentration of 0.001–1000 μg/ml) were added, giving a final volume of 200 μl (tetraplicate wells) and incubated for 48 h at 37°C under 5% CO_2_ condition.

### Cell proliferation assay

Cells were treated with different concentrations of barberry extracts (0.001–1000 μg/ml). After 48 h of incubation, cell proliferation was measured based on the MTT (3-(4,5-dimethylthiazole-2-yl)-2,5-diphenyl tetrazolium bromide) reduction assay [12]. When MTT reacts with mitochondrial dehydrogenase in living cells, the blue fromazan crystals will be formed. Procedure in brief: 20 μl of MTT (5 mg/ml in PBS) was added to 200 μl wells (in one tenth of the total volume) and incubated for 4 h at 37°C and 5% CO_2_. After incubation the medium was removed and the formazan blue crystals were dissolved by 100 μl of acidic isopropanol (0.04 M HCl in isopropanol). By using Multiskan MS microplate reader (Thermo lab systems, USA) at 540 nm each well was read. The result of the test was expressed as a stimulation index (SI), which is optical density at 540 nm (OD540) of the test samples/OD540 of negative control.

### Cytokine ELISA

The levels of IFN-γ, IL-4, IL-10 and TGF-β cytokines in culture supernatant were measured using an ELISA kit (eBioscience, Germany), according to the manufacturer's instructions. All samples were measured at least two times.

### Data analysis

Statistical analysis was performed using SPSS version 15. One-way analysis of variance (ANOVA) was done to analyze data and followed by Fisher's least significant difference (LSD) test. The p-values less than 0.05 were considered to indicate significant differences. Results are expressed as a mean ± standard deviation (SD).

## Results

### Lymphocytes subtypes’ proliferation

Proliferation assay of PHA-stimulated, LPS-stimulated and unstimulated spelnocytes treated with various concentrations of barberry alcoholic and aqueous extracts are shown in Figures 1 & 2; 1–1000 μg/ml of alcoholic extract reduced PHA-stimulated splenocytes (as T cells) proliferation and enhanced proliferation of LPS-stimulated (as B cells) and unstimulated splenocytes (p < 0.05); 0.01–1000 μg/ml of aqueous extract reduced PHA-stimulated splenocytes (as T cells) proliferation (p < 0.05). 0.001–1 μg/ml of aqueous extract reduced LPS-stimulated splenocytes (as B cells) proliferation and 1000 μg/ml increased these cells proliferation (p > 0.05); 1000 μg/ml of aqueous extract increased unstimulated splenocytes proliferation (p > 0.05). Significant differences are designated as p < 0.05.

### Cytokine production

Cytokine assay on *B. integerrima* treated balb/c splenocytes supernatant (Figures 3–6) showed that 0.001–1000 μg/ml of both extracts affected splenocytes cytokine production.

#### IFN-γ

Both *B. integerrima* alcoholic and aqueous extracts in the range of 0.001–1000 μg/ml downregulated IFN-γ production Figure 3. 0.01–1000 μg/ml of alcoholic extract and 1–1000 μg/ml of aqueous extract effect on IFN-γ release were statistically significant (p < 0.05). Optimum dose for alcoholic extract was 1 μg/ml and for aqueous extract was 1000 μg/ml (control group vs 0.001–1000 µg/ml of both *B. integerrima* aqueous and alcoholic extracts).

#### IL-4

0.1–1000 μg/ml of *B. integerrima* concentration of both alcoholic and aqueous extracts induced IL-4 production and 1 μg/ml concentration of both extracts are optimum dose (p < 0.05). IL-4 levels were increased about one-and-a-half-fold by both alcoholic and aqueous extracts in the range of 0.1–1000 µg/ml of untreated splenocytes versus 0.1–1000 µg/ml ingredients affected splenocytes (Figure 4).

#### IL-10

IL-10 production was enhanced by 10–1000 μg/ml of alcoholic extract and 100–1000 μg/ml of aqueous extract (p < 0.05). Both extracts also had optimum stimulatory effect on IL-10 production in 1000 µg/ml (Figure 5).

#### TGF-β

Splenocytes produced much more TGF-β as they were treated by 0.001–1000 μg/ml of alcoholic extract and 0.1–1000 μg/ml of aqueous extract (Figure 6). Optimum doses of extracts in enhancing TGF-β production was 100 μg/ml of alcoholic extract and 10 μg/ml of aqueous extract. The strongest effect of *integerrima* extract on splenocytes has been shown by TGF-β production in which production of this cytokine was two to three times more than normal group (untreated splenocytes vs 0.001–1000 µg/ml ingredients affected splenocytes).

## Discussion

Although pharmacological investigations of barberry have been reported by many other researchers in the past [12,13], there is big interest in barberry because of its reported beneficial effect in immune disorders and suppression of the immune system especially lymphocytes. One of the effective constituents of barberry is berberine, which alone or in combination with other pharmaceutically active compounds have applications in various therapeutic areas such as cancer, inflammation, diabetes, depression, hypertension and various infectious areas [14]. In this study, we evaluated alcoholic and aqueous *B. integerrima* extracts effects on mitogen-stimulated lymphocytes (T and B cells) expansion and their cytokine production profile. Exploration of lymphocyte proliferation showed that higher concentrations (1–1000 μg/ml) of *B. integerrima* alcoholic and aqueous extracts have a suppressive effect on PHA-stimulated splenocytes (as T cells) proliferation and 1–1000 μg/ml of alcoholic extracts increased LPS-stimulated (as B cells) and unstimulated splenocytes expansion (Figures 1 & 2).

In brief, our results have shown that *B. integerrima* ingredients in both water solution and alcohol solution, suppress T-cell proliferation and enhance B-cell expansion. The suppressive effects of barberry in cell growth have been studied many times and it has been found that barberry can be used in inhibition of cancer cell growth [11,15]. *B. integerrima* ingredients can also change the cytokine production pattern in lymphocytes; our research results showed that some concentration of *B. integerrima* both extracts, especially 1–1000 μg/ml can reduce IFN-γ release (Figure 3) and this reduction may contribute in suppression of T-cell expansion. In this study,*B. integerrima* both extracts in some ranges (1–1000 μg/ml) increased IL-4 release (Figure 4) may be connected to the expansion of B cells as IL-4 is major B cell growth factor. IL-4 promotes the development of Th2 cells and allergic inflammation so IL-4 increase may exacerbate ongoing allergy but can be suppressed by IL-10 and TGF-β regulatory cytokines; still further studies are needed to investigate *B. integerrima* influence on different types of allergy.

Some interesting data are that, in our research, *B. integerrima* had impressive positive effect on TGF-β production. Both alcoholic and aqueous extracts increase the level of IL-4, IL-10 and TGF-β (Figures 4, 5 & 6); due to TGF-β and IL-10 regulatory and suppressive function the cells environment can't shift to inflammatory condition and shifts toward anti-inflammatory condition.

A 10–1000 μg/ml of both alcoholic and aqueous extracts increase TGF- β, IL-4 and IL-10 production; these changes in cytokine release pattern may result in T lymphocytes shift toward Th2 or regulatory T cell.

Lin *et al*. have shown the same results in cytokine production changes made by barberry ingredients as our research; some appropriate concentration of barberry upregulated IL-4 and IL-10 expression and by that may have immunomodulatory activity, which suggested that barberry may shift the Th1/Th2 balance toward Th2 polarization *in vitro* [10]. Also, another study showed that barberry ingredients were found to promote STAT4 degradation, resulting in a significant reduction in IFN-γ T cells [16]. Another study investigated anti-inflammatory effects of barberry ingredients supplementation *in vivo* using a NOD mouse model, suggesting that barberry supplementation decreased the expression ratios of Th1/Th2 cytokines and reduced proinflammatory cytokines [17]. In addition to these findings, there are some other studies that show barberry ingredients could affect immune responses, for instance a study of barberry contents showed inhibition of NO production and inducible nitric oxide synthase (iNOS) expression in a dose-dependent manner in LPS-stimulated murine macrophages [18]. Therefore, our research and others indicate that barberry modulates T-cell expansion and functions; moreover, it can restore cellular immunity, which would have beneficial applications.

## Conclusion

In conclusion, our study showed that *B. integerrima* ingredients are capable of suppressing T-cell proliferation and B-cell expansion, which is compatible with previous studies for reduction of cellular T-cell responses and shift toward Th2 functions. A survey on cytokine release showed reduction of IFN-γ release (Th1 cytokine) which is proinflammatory mediator, and an increase of IL-4, IL-10 and TGF-β production. It also significantly increases TGF-β production which is anti-inflammatory cytokine. Other studies showed the same anti-inflammatory functions of barberry contents. As a result barberry could suppress T cells and their functions and enhance B cells expansion, function and humoral responses. *B. integerrima* influences cytokine profile and modifies the pattern and shift responses toward Th2.

## Future perspective

Relation between food and immune system is going to be even more important for researchers because mankind's relationship with food may take on different forms in the future, which are very difficult to predict. The way people eat is related to the immune system development and answers in many conditions including allergy, cancer and fertility, *etc*. Researchers are going to study every component of different herb and food in order to find potential answers for many challenging questions in immune responses and replace many chemical drugs with food and herbs.

**Executive summary**

**Introduction**

*Berberis integerrima* is worldwide used in traditional medicine for the treatment of various ailments.
Barberry has diverse effects on the immune system.
One of the effective constituents of barberry is berberine, it has immunomodulatory function, decreasing Th17 and Th1 cell differentiation and changing cytokine production.
In this study the effect of *B. integerrima* aqueous and alcoholic extracts were investigated on mice splenocytes, as T and B cells are the major effector cells in cellular and humoral immune responses.

**Materials & methods**

Balb/c mice splenocytes were treated with phytohemagglutinin (PHA), lypopolysaccharide (LPS) mitogens and 0.001–1000 µg/ml concentrations of *B. integerrima* alcoholic and aqueous extracts.
Cell proliferation was measured based on MTT (3-(4, 5-dimethylthiazole-2-yl) -2, 5-diphenyl tetrazolium bromide) assay.
Levels of IFN-γ, IL-4, IL-10 and TGF-β cytokines in culture supernatant were measured using ELISA.
Statistical analysis was performed using SPSS, the p-values <0.05 were considered to indicate significant differences.

**Results**

1–1000 µg/ml of alcoholic extract reduced PHA-stimulated splenocytes (as T cells) proliferation and enhanced proliferation of LPS-stimulated (as B cells) and unstimulated cells (p < 0.05).
1–1000 µg/ml of aqueous and alcoholic extracts reduced PHA stimulated splenocytes proliferation (p < 0.05).
1–1000 µg/ml of both *B. integerrima* extracts suppressed IFN-γ production (p < 0.05).
0.1–1000 µg/ml of both alcoholic and aqueous extracts enhanced IL-4 production in splenocytes (p < 0.05).
100–1000 µg/ml of both *B. integerrima* extracts increased IL-10 production (p < 0.05).
0.1–1000 µg/ml of both extracts enhanced TGF-β production from splenocytes (p < 0.05).

**Discussion**

There is renewed interest in barberry because of its beneficial effect in immune disorders and suppression of the immune system especially T lymphocytes.
One of the effective constituents of *B. integerrima* is berberine which has applications in various therapeutic areas.
In this study, we evaluated *B. integerrima* alcoholic and aqueous extracts effect on mitogen (PHA, LPS) stimulated lymphocytes (T and B cells) expansion and their cytokine production profile.
*B. integerrima* ingredients in both water and alcohol solution, suppress T-cell proliferation and enhance B-cell expansion.
*B. integerrima* extracts increased IL-4 and IL-10 release and IL-4 is connected to expansion of B cells.
*B. integerrima* extracts reduced IFN-γ release and it could be related to suppress of T-cell expansion.
*B. integerrima* both extracts had impressive increasing effect on TGF-β production.
Increasing of IL-4, IL-10 and TGF- β and decrease of IFN-γ could be related to shift the responses towards Th2.

**Conclusion**

*B. integerrima* could suppress T cells and their functions and enhance B-cell expansion, function and humoral responses.
*B. integerrima* changes cytokine profile and shift responses toward Th2.
*B. integerrima* components act as anti-inflammatory in immune responses.
